# Lessons learnt from implementation of the International Health Regulations: a systematic review 

**DOI:** 10.2471/BLT.16.189100

**Published:** 2017-12-11

**Authors:** Amitabh B Suthar, Lisa G Allen, Sara Cifuentes, Christopher Dye, Jason M Nagata

**Affiliations:** a South African Centre for Epidemiological Modelling and Analysis, University of Stellenbosch, Private Bag X1, Matieland, Stellenbosch, 7602, South Africa.; b TMF Health Quality Institute, Austin, United States of America (USA).; c Center for Public Health Initiatives, University of Pennsylvania, Philadelphia, USA.; d Department of Strategy, Policy and Information, World Health Organization, Geneva, Switzerland.; e Department of Pediatrics, University of California San Francisco, San Francisco, USA.

## Abstract

**Objective:**

To respond to the World Health Assembly call for dissemination of lessons learnt from countries that have begun implementing the International Health Regulations, 2005 revision; IHR (2005).

**Methods:**

In November 2015, we conducted a systematic search of the following online databases and sources: PubMed®, Embase®, Global Health, Scopus, World Health Organization (WHO) Global Index Medicus, WHO Bulletin on IHR Implementation and the International Society for Disease Surveillance. We included identified studies and reports summarizing national experience in implementing any of the IHR (2005) core capacities or their components. We excluded studies that were theoretical or referred to IHR (1969). Qualitative systematic review methodology, including meta-ethnography, was used for qualitative synthesis.

**Findings:**

We analysed 51 articles from 77 countries representing all WHO Regions. The meta-syntheses identified a total of 44 lessons learnt across the eight core capacities of IHR (2005). Major themes included the need to mobilize and sustain political commitment; to adapt global requirements based on local sociocultural, epidemiological, health system and economic contexts; and to conduct baseline and follow-up assessments to monitor the status of IHR (2005) implementation.

**Conclusion:**

Although experiences of IHR (2005) implementation covered a wide global range, more documentation from Africa and Eastern Europe is needed. We did not find specific areas of weakness in monitoring IHR (2005); sustained monitoring of all core capacities is required to ensure effective systems. These lessons learnt could be adapted by countries in the process of meeting IHR (2005) requirements.

## Résumé

**Objectif:**

Répondre à la demande de l'Assemblée mondiale de la Santé de diffuser les leçons tirées par les pays qui ont commencé à mettre en œuvre le Règlement sanitaire international, tel que révisé en 2005 – RSI (2005).

**Méthodes:**

En novembre 2015, nous avons effectué une recherche systématique dans les bases de données et sources en ligne suivantes: PubMed®, Embase®, Global Health, Scopus, l'Index Medicus mondial de l'Organisation mondiale de la Santé (OMS), le bulletin de l'OMS sur la mise en œuvre du RSI et l'International Society for Disease Surveillance. Nous avons inclus des études et des rapports résumant une expérience nationale de mise en œuvre de l'une des principales capacités du RSI (2005) ou de ses composants. Nous avons exclu les études théoriques ou qui se référaient à l'édition du RSI de 1969. Nous avons utilisé une méthodologie qualitative de la revue systématique, et notamment une méta-éthnographie, pour réaliser une synthèse qualitative.

**Résultats:**

Nous avons analysé 51 articles qui provenaient de 77 pays représentant toutes les régions de l'OMS. Les méta-synthèses ont mis en évidence 44 leçons tirées des huit principales capacités du RSI (2005). Les thèmes centraux reflétaient la nécessité de susciter et maintenir l'engagement politique; d'adapter les exigences mondiales en fonction du contexte socioculturel, épidémiologique, du système de santé et économique local; et de réaliser des évaluations de référence et de suivi pour surveiller l'avancée de la mise en œuvre du RSI (2005).

**Conclusion:**

S'il est vrai que les expériences de mise en œuvre du RSI (2005) ont couvert une vaste zone géographique, une plus ample documentation provenant d'Afrique et d'Europe de l'Est est nécessaire. Nous n'avons pas trouvé de points faibles spécifiques lors du suivi du RSI (2005); un suivi permanent de l'ensemble des principales capacités est indispensable pour garantir l'efficacité des systèmes. Ces leçons tirées pourraient être adaptées par les pays engagés dans un processus de respect des exigences du RSI (2005).

## Resumen

**Objetivo:**

Responder a la convocatoria de la Asamblea de la Organización Mundial de la Salud para difundir las lecciones aprendidas de los países que han empezado a aplicar el Reglamento Sanitario Internacional, revisión de 2005; RSI (2005).

**Métodos:**

En noviembre de 2015, se realizó una búsqueda sistemática de las siguientes fuentes y bases de datos en línea: PubMed®, Embase®, Global Health, Scopus, Global Index Medicus de la Organización Mundial de la Salud (OMS), el Boletín de la OMS sobre la implementación del RSI y la Sociedad Internacional para la Vigilancia de Enfermedades. Se incluyeron estudios e informes identificados que resumen la experiencia nacional de la implementación de cualquiera de las capacidades básicas del RSI (2005) o sus componentes. Se excluyeron los estudios teóricos o que hicieran referencia al RSI (1969). Se usó una metodología cualitativa de revisión sistemática, incluida la metaetnografía, para la síntesis cualitativa.

**Resultados:**

Se analizaron 51 artículos de 77 países que representan a todas las Regiones de la OMS. Las metasíntesis identificaron un total de 44 lecciones aprendidas en las ocho capacidades básicas del RSI (2005). Los temas principales incluyeron la necesidad de movilizar y mantener el compromiso político; de adaptar los requisitos globales en función de los contextos socioculturales, epidemiológicos, del sistema de salud y económicos locales; y de realizar evaluaciones iniciales y de seguimiento para supervisar el estado de la implementación del RSI (2005).

**Conclusión:**

Aunque las experiencias de la implementación del RSI (2005) cubrieron un amplio rango global, hace falta más documentación de África y Europa del Este. No se hallaron áreas específicas de debilidad en la supervisión del RSI (2005). Es necesaria una supervisión sostenida de todas las capacidades básicas para asegurar unos sistemas efectivos. Estas lecciones aprendidas podrían ser adaptadas por los países en proceso de cumplir con los requisitos del RSI (2005).

## ملخص

**الغرض:**

الاستجابة إلى نداء جمعية الصحة العالمية من أجل نشر الدروس المستفادة من البلدان التي بدأت في تنفيذ اللوائح التنظيمية الصحية الدولية، مراجعة 2005؛ اللوائح التنظيمية الصحية الدولية الصادرة لعام (2005).

**الطريقة:**

قمنا في نوفمبر/تشرين الثاني من عام 2015 بإجراء بحث منهجي لقواعد البيانات المتواجدة على شبكة الإنترنت والمصادر التالية: PubMed®‎، وEmbase®‎، وGlobal Health، وScopus، والفهرس الطبي العالمي التابع لمنظمة الصحة العالمية، ومجلة منظمة الصحة العالمية بشأن تنفيذ اللوائح التنظيمية الصحية الدولية، والمجتمع الدولي لمراقبة الأمراض. وقمنا بتضمين الدراسات والتقارير المحددة التي تلخص التجربة الوطنية في تنفيذ أي من الإمكانيات الأساسية للوائح التنظيمية الصحية الدولية (لعام 2005) أو مكوناتها. وقمنا باستبعاد الدراسات الافتراضية أو التي تشير إلى اللوائح التنظيمية الصحية الدولية (لعام 1969). وقد تم استخدام نهج المراجعة المنهجية النوعية، بما في ذلك الدراسة التجميعية للتراث العرقي لتكوين النهج النوعي.

**النتائج:**

قمنا بتحليل 51 مقالة صادرة من 77 بلدًا تمثل جميع المناطق الخاضعة لسلطة منظمة الصحة العالمية. وقام التكوين التجميعي بتحديد إجمالي 44 درسًا مستفادًا عبر الإمكانيات الأساسية الثمانية للوائح التنظيمية الصحية الدولية (لعام 2005). واشتملت الموضوعات الكبيرة على الحاجة إلى حشد ودعم الالتزام السياسي لمواءمة المتطلبات العالمية بناءً على السياقات الاجتماعية والثقافية، والوبائية، والنظام الصحي، والاقتصادية؛ وإجراء تقييمات لخط الأساس والمتابعة لمراقبة حالة تنفيذ اللوائح التنظيمية الصحية الدولية (لعام 2005).

**الاستنتاج:**

بالرغم من أن تجارب تنفيذ اللوائح التنظيمية الصحية الدولية (لعام 2005) قد شملت نطاقًا عالميًا واسعًا، فإن هناك حاجة إلى إجراء المزيد من التوثيق من أفريقيا وأوروبا الشرقية. ولم نجد مظاهر ضعف محددة عند مراقبة اللوائح التنظيمية الصحية الدولية (لعام 2005)؛ المراقبة المستدامة لجميع الإمكانيات الأساسية مطلوبة لضمان تكوين أنظمة فعالة. يمكن مواءمة هذه الدروس المستفادة من جانب البلدان في أثناء عملية تلبية متطلبات اللوائح التنظيمية الصحية الدولية (لعام 2005).

## 摘要

目标 为响应世界卫生大会呼吁，关注已经开始实施 2005 年修订版《国际卫生条例》国家的经验教训。

**方法:**

2015 年 11 月，我们对以下在线数据库和信息来源进行了系统检索：PubMed®、Embase®、全球健康、Scopus、世界卫生组织全球医学索引、世界卫生组织关于《国际卫生条例》实施的公报和国际疾病监控学会。我们列入了确定的研究和报告，这些研究和报告总结了实施《国际卫生条例 (2005)》时的任一核心能力或其组成部分的国家经验。我们排除了理论上或称为《国际卫生条例 (1969)》的研究。包括元人种学在内的定性系统评价方法被用于定性综合集成法。

**发现:**

我们分析了代表所有世界卫生组织地区的 77 个国家的 51 篇文章。综合分析确定了《国际卫生条例 (2005)》的八项核心能力，总共获得了 44 项经验教训。主要议题包括需要调动和维持政治承诺；根据当地的社会文化、流行病学、卫生系统和经济环境调整全球要求；并开展基线和后续评估，以监测《国际卫生条例 (2005)》实施情况。

**结论:**

虽然《国际卫生条例 (2005)》的实施经验涵盖了较为广泛的全球范围，但仍需要来自非洲和东欧的更多文件。我们在监测《国际卫生条例 (2005)》实施情况时，并没有发现具体的弱点区域，需要对所有核心能力进行持续监测，以确保有效的系统。各国在达到《国际卫生条例 (2005)》要求的过程中可以根据这些经验教训进行调整。

## Резюме

**Цель:**

Откликнуться на призыв Всемирной ассамблеи здравоохранения распространить информацию о практическом опыте стран, которые приступили к реализации Международных медико-санитарных правил в редакции 2005 года; ММСП (2005) (IHR (2005)).

**Методы:**

В ноябре 2015 года авторы провели систематический поиск в следующих электронных базах данных и источниках: PubMed®, Embase®, Global Health, Scopus, Глобальной базе данных Global Index Medicus Всемирной организации здравоохранения (ВОЗ), Бюллетене ВОЗ, посвященном реализации ММСП, и Международном обществе по надзору за заболеваниями (International Society for Disease Surveillance). В обзор включены найденные исследования и отчеты, обобщающие национальный опыт реализации каких-либо основных возможностей ММСП (2005) или их компонентов. Исследования, которые были теоретическими или относились к ММСП (1969), были исключены из обзора. Для качественного синтеза была использована качественная методология систематического обзора, включая метаэтнографию.

**Результаты:**

Авторы провели анализ 51 статьи из 77 стран, представляющих все регионы ВОЗ. При метасинтезе было выявлено в общей сложности 44 примера опыта, полученного при реализации восьми основных возможностей ММСП (2005). Основные темы включали необходимость мобилизации и поддержания стратегической приверженности, адаптации глобальных потребностей с учетом местных социокультурных, эпидемиологических, экономических условий и уровня системы здравоохранения, а также проведения исходной и последующих оценок для мониторинга хода реализации ММСП (2005).

**Вывод:**

Хотя опыт реализации ММСП (2005) охватывал широкий глобальный диапазон, требуется дополнительная документация из Африки и Восточной Европы. Авторы не обнаружили определенных слабых мест при мониторинге ММСП (2005); для обеспечения эффективной работы систем требуется постоянный мониторинг всех основных возможностей. Этот полученный опыт может быть адаптирован странами при выполнении требований ММСП (2005).****

## Introduction

While bi- and multilateral communication and collaboration are the foundation for global control of infectious disease epidemics, they are strengthened by the International Health Regulations (IHR). First introduced in 1969, IHR is global legislation requiring countries to link and coordinate specific actions.^1^ These regulations were originally designed to control cholera, plague, yellow fever, smallpox, relapsing fever and typhus. Given the need to expand the scope to include new epidemics and to improve global coordination, the regulations were revised in 2005, as IHR (2005).^2^ One of the key changes was the requirement for countries to notify the World Health Organization (WHO) of all events that may constitute a public health emergency of international concern and to respond to requests for verification of information about these events.^2^ Since IHR (2005) was adopted by WHO States’ Parties, several outbreaks, epidemics and pandemics have been declared public health emergencies of international concern: the H1N1 pandemic influenza in 2009, wild poliovirus in 2014, Ebola virus disease in 2014, and Zika virus and increases in neurological disorders and neonatal malformations in 2016.^3^^–^^6^

IHR (2005) also requires all countries to develop, strengthen and maintain eight core public health capacities.^2^^,^^7^ These core capacities cover: (i) national legislation, policy and financing; (ii) coordination and national focal point communications; (iii) surveillance; (iv) response; (v) preparedness; (vi) risk communication; (vii) human resources; and (viii) laboratory.^8^ Given varying levels of health and socioeconomic development across countries, there have been challenges in implementing these requirements. By the original deadline of June 2012, only 42 (22%) of the 192 WHO Member States had met the core capacity requirements.^9^ Yet substantial progress has been made in some areas. These include establishing a 24-hour presence of a national focal point to communicate with all relevant sectors within government, all national stakeholders and WHO; increased transparency in reporting events; using early warning systems more systematically; and better communication and collaboration between the animal and human health sectors. Nevertheless, by the end of 2015, 127 of the 192 countries had not meet all IHR (2005) core capacity requirements.^9^^,^^10^

Although IHR (2005) describes what must be achieved by countries, there is limited knowledge on how countries should proceed in achieving the core capacities. To fill this gap and accelerate implementation of IHR (2005), the World Health Assembly in 2015 identified a need to evaluate and share the lessons learnt from countries that have implemented IHR (2005).^11^ While country exchanges and regional meetings are one mechanism to achieve this, we systematically evaluated published literature and reports for lessons learnt from national experience in implementing IHR (2005).

## Methods

This systematic review was conducted in accordance with Enhancing Transparency in Reporting the Synthesis of Qualitative Research guidelines, using a predefined protocol (International Prospective Register of Systematic Reviews identification number: CRD42016038719).^12^^,^^13^ We systematically searched the PubMed®, Embase®, Global Health, Scopus and WHO Global Index Medicus databases without any limitations of language, publication type or date on 13 November 2015. We searched all databases for the terms “IHR” or “International Health Regulations” to ensure we captured articles focusing specifically on IHR implementation. We did not search additional terms for the core capacities since this would decrease the number of results from the databases and potentially decrease the sensitivity of the search strategy. We also searched updates from the WHO bulletin on IHR implementation and abstracts from the International Society for Disease Surveillance annual conferences.^14^^,^^15^ Studies were included when they summarized national experience in implementing any of the IHR (2005) core capacities or their components (Box 1).^8^ We excluded studies that referred to IHR (1969) or were theoretical and not rooted in national experience.

Box 1 **Core capacities and components of the International Health Regulations (2005)**

**Core capacity 1: National legislation, policy and financing**

Component 1A: National legislation and policy

Component 1B: Financing

**Core capacity 2: Coordination and national focal point communications**

Component 2A: IHR coordination, communication and advocacy

**Core capacity 3: Surveillance**

Component 3A: Indicator-based surveillance

Component 3B: Event-based surveillance

**Core capacity 4: Response**

Component 4A: Rapid response capacity

Component 4B: Case management

Component 4C: Infection control

Component 4D: Disinfection, decontamination and vector control

**Core capacity 5: Preparedness**

Component 5A: Public health emergency preparedness and response

Component 5B: Risk and resource management for IHR preparedness

**Core capacity 6: Risk communication**

Component 6A: Policy and procedures for public communications

**Core capacity 7: Human resources**

Component 7A: Human resource capacity

**Core capacity 8: Laboratory**

Component 8A: Policy and coordination of laboratory services

Component 8B: Laboratory diagnostic and confirmation capacity

Component 8C: Laboratory biosafety and laboratory biosecurity (biorisk management)

Component 8D: Laboratory-based surveillance

IHR: International Health Regulations.Source: International Health Regulations (2005).^7^

Two authors independently screened all abstracts, articles and reports and then matched the full texts selected during screening against the inclusion criteria. The reference lists of relevant articles and reviews were also searched for additional studies and reports. Articles meeting the inclusion criteria were included in the review. Three authors completed the data extraction using standardized extraction tables.

Two authors independently read and assessed the quality of included studies using the Critical Appraisal Skills Programme qualitative research checklist.^16^ Disagreements in quality assessment between reviewers were resolved through discussion. In case new insights, grounded in data, might be generated in studies classified as low methodological quality, no studies were excluded on the basis of the quality assessment.^17^^,^^18^

We used the Cochrane qualitative systematic review method, including meta-ethnography, to synthesize qualitative data.^17^^,^^19^^–^^22^ Meta-ethnography involved three steps: (i) reciprocal translational analysis (comparison); (ii) refutational synthesis (contrast); and (iii) line of argument synthesis (high-level synthesis).^23^^,^^24^ We extracted key quotations from each study into an Excel spreadsheet (Microsoft Corporation, Redmond, United States of America) and organized these into facilitators or barriers to implementation for each component of the eight IHR core capacities.^25^ Each quotation was summarized into a theme. For reciprocal translation analysis, we compared similar themes from individual studies within each core capacity and synthesized lessons learnt that reflected themes from multiple studies. For refutational synthesis, we contrasted themes between individual studies within each core capacity and noted disagreements. Line of argument synthesis was used to identify lessons learnt that were relevant to multiple core capacities (i.e. cross-cutting themes).^24^

## Results

### Search results

**Fig. 1:**
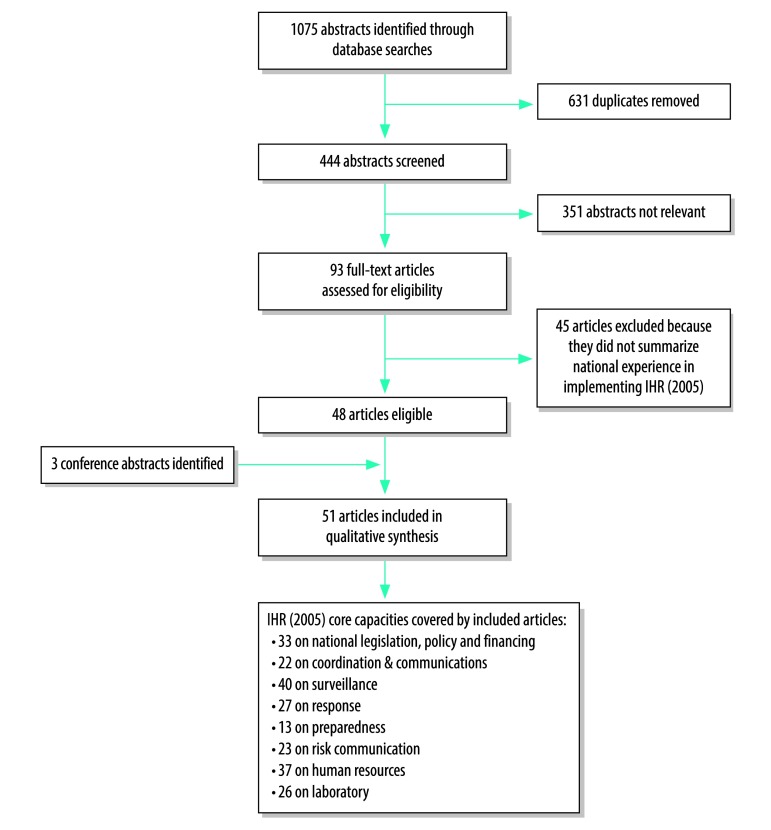
Flowchart on the selection of articles for the systematic review on the implementation of the International Heath Regulations (2005)

**Fig. 2:**
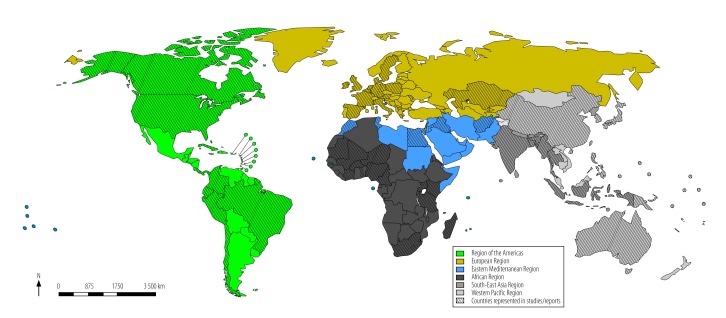
Country location of studies and reports included in the systematic review of implementation of the International Heath Regulations (2005), by World Health Organization Region

We identified 1075 abstracts through database searches. After removing duplicates and screening out non-relevant abstracts, we assessed 93 full-text articles for eligibility and excluded 45. We identified three abstracts from the International Society for Disease Surveillance’s annual conferences. In total 51 articles, published from 2007 to 2015,^26^^–^^77^ met the eligibility criteria (Fig. 1; Table 1 and Table 2 (both available at: http://www.who.int/bulletin/volumes/96/2/16-189100). A more detailed summary of the included articles and themes identified is available from the corresponding author. Some articles reported national experience in IHR (2005) implementation in multiple countries. In total, 77 countries were represented from all WHO Regions: 23 from Western Pacific, 16 from Europe, 14 from Africa, 11 from the Americas, eight from Eastern Mediterranean and five from South-East Asia (Fig. 2). A total of 44 lessons learnt were synthesized from the eligible articles (Box 2; Box 3).

IHR (2005): International Health Regulations, 2005 revision.

Notes: Some of the included articles did not report on all eight core capacities of the IHR (2005). The three abstracts were from annual conferences of the International Society for Disease Surveillance.

**Table 1:** Summary of articles included in the systematic review of the implementation of the International Health Regulations (2005)

Author, year	Data collection year(s)	Publication year	Setting	WHO Region	Study design	Quality assessment score (out of 10)^a^	IHR core capacities reported^b^
Calain, 2007^30^	Not reported	2007	Cambodia, Lao People's Democratic Republic, India and Uganda	Africa, South-East Asia and Western Pacific	Case study of the alternative view of donor-driven surveillance building	N/A	1, 2, 3, 4, 7, 8
Lyons et al., 2007^53^	2007	2007	Tunisia	Eastern Mediterranean	Evaluation of surveillance systems to identify implications of IHR (2005) implementation	8	1, 3, 4, 6, 7, 8
Narain et al., 2007^57^	2007	2007	India	South-East Asia	Evaluation of the status of implementation of IHR core capacities	N/A	1, 2, 3, 6, 7, 8
Perry et al., 2007^60^	Not reported	2007	Africa, Central America and Philippines	Africa, Americas and Western Pacific	Qualitative study of surveillance objectives, surveillance case definitions, action thresholds and recommendations for 19 priority infectious diseases	8	1, 3, 4
Kicman-Gawlowska, 2008^48^	Not reported	2008	Poland	Europe	Evaluation of coordination, surveillance and communication in transitioning to IHR (2005) requirements	N/A	1, 2, 3, 4, 6
Kicman-Gawlowska, 2009^49^	2007–2009	2009	Poland	Europe	Evaluation of the national focal point and status of implementation of IHR (2005)	N/A	2, 5, 7
May et al., 2009^55^	2009	2009	Australia, Bangladesh, Burkina Faso, Costa Rica, China, Taiwan, Côte d’Ivoire, Egypt, Eritrea, French Guiana, Indonesia, Jamaica, Lao People's Democratic Republic, Peru, Solomon Islands, United Republic of Tanzania, Uganda, USA and Vanuatu	Africa, Americas, Eastern Mediterranean, South-east Asia and Western Pacific	Qualitative study of the application of syndromic surveillance to IHR (2005)	N/A	3, 6
Patel & Phillips, 2009^58^	2007–2009	2009	Australia	Western Pacific	Evaluation of different educational and training models and lessons learnt	N/A	3, 7
Stikova et al., 2010^68^	Not reported	2010	The former Yugoslav Republic of Macedonia	Europe	Case study of syndromic surveillance	N/A	1, 2, 3, 6, 7, 8
Takahashi-Omoe & Omoe, 2009^70^	Not reported	2009	Japan	Western Pacific	Evaluation of the regulatory framework for preventing zoonoses in Japan	N/A	1, 2
Van Vliet et al., 2009^72^	2009	2009	Netherlands	Europe	Case study describing a new public health act regarding notification of communicable diseases and what will change for providers	N/A	1, 7
Chretien et al., 2010^31^	2009	2010	Afghanistan	Eastern Mediterranean	Evaluation of challenges to implementing IHR (2005) by countries with active conflicts, and proposed role of coordinated international military in supporting IHR (2005)	N/A	1, 2, 3, 4, 7, 8
Johns & Blazes, 2010^42^	2008–2009	2010	All locations with USA Department of Defence staff	Africa, Americas, South-East Asia, Europe, Western Pacific and Eastern Mediterranean	Evaluation of the Department of Defence’s work in capacity-building in all WHO regions	N/A	2, 3, 4, 6, 7, 8
Kandun et al., 2010^44^	2007–2009	2010	Indonesia	South-East Asia	Qualitative study of the national Field Epidemiology Training Programme work plan based on the existing programme and projected human resource needs	N/A	1, 7
Kant & Krishnan, 2010^45^	2010	2010	India	South-East Asia	Evaluation of the status and progress of IHR (2005) implementation	N/A	3, 4, 5, 6, 7, 8
Katz & Kornblet, 2010^47^	2007–2010	2010	Australia, Canada, Germany and India	Americas, South-East Asia, Europe and Western Pacific	Assessment of IHR (2005) implementation strategies	7	1, 2, 3, 5
Masanza et al., 2010^54^	2007–2010	2010	Burkina Faso, Kenya, Mali, Niger Nigeria, Rwanda, South Africa, Togo and United Republic of Tanzania	Africa	Case study of the African Field Epidemiology Network	N/A	1, 3, 4, 7, 8
Wamala et al., 2010^73^	2009	2010	Uganda	Africa	Qualitative study of national IHR (2005) core capacities	8	1, 2, 3, 4, 5, 6, 7, 8
Gomez et al., 2011^37^	Not reported	2011	Colombia, Panama, Ecuador, Brazil and Peru	Americas	Qualitative study of regional collaboration for achieving IHR (2005) core capacities and the Millennium Development Goals based on the review of documents and information provided by key stakeholders in this field	8	3, 7
Mmbuji et al., 2011^56^	2007–2011	2011	United Republic of Tanzania	Africa	Case study of the Field Epidemiology and Laboratory Training Programme and progress in increasing capacity for surveillance and laboratory core capacities	N/A	1, 2, 4, 5, 7, 8
Quandelacy et al., 2011^62^	2010	2011	Djibouti, France, French Guiana, Peru, Philippines and USA	Americas, Eastern Mediterranean, Europe and Western Pacific	Case study of a conference to gather and share experiences of surveillance capacity-building and IHR (2005) implementation	N/A	3, 4, 7, 8
Aguilera et al., 2012^26^	Not reported	2012	Chile	Americas	Evaluation of progress made in implementing IHR (2005)	N/A	2, 3, 4, 5, 6, 7, 8
Gala et al., 2012^35^	2011	2012	Cuba	Americas	Qualitative study of an instrument evaluating basic capacities such as legislation and response to public health emergencies	8	1, 3, 4, 5, 6, 7
Harutyunyan, 2012^40^	2012	2012	West Bank and Gaza Strip	Eastern Mediterranean	Evaluation of progress and challenges in implementation of IHR (2005)	N/A	1
Kool et al., 2012^28^	2010–2012	2012	Pacific Island States and Territories^c^	Western Pacific	Qualitative study describing experience with syndromic surveillance for early warning indicators	8	2, 3, 4, 6, 8
Paterson et al., 2012^59^	2011	2012	Five Pacific Island States and Territories^d^	Western Pacific	Case study of the syndromic surveillance system and analysis of syndromic data reported to WHO by all participating territories	8	1, 2, 3, 6, 7
Rajatonirina et al., 2012^63^	2007–2011	2012	Madagascar	Africa	Case study of the surveillance system	NA	2, 3, 6, 7
Teixeira et al., 2012^71^	2008–2009	2012	Brazil	Americas	Qualitative study to assess health system surveillance structure and surveillance and response procedures regarding compliance with IHR (2005)	8	1, 2, 3, 4, 6, 7, 8
Bakari & Frumence, 2013^27^	2013	2013	United Republic of Tanzania	Africa	Qualitative study of national IHR (2005) implementation using in-depth interviews, focus group discussions and document reviews	8	1, 2, 3, 4, 5, 6, 7, 8
Craig et al., 2013^32^	2012	2013	Pacific Island States and Territories^e^	Western Pacific	Evaluation of a tailored tool to meet obligations to IHR (2005)	N/A	3, 4, 5, 8
Dagina et al., 2013^33^	2009–2012	2013	Papua New Guinea	Western Pacific	Evaluation of the performance of the event-based surveillance system	8	2, 3, 4, 6, 7, 8
Friede, 2013^34^	2012	2013	Cambodia, China, Israel, Jordan, Lao People's Democratic Republic, Myanmar, Thailand, USA, Viet Nam and West bank and Gaza Strip	Americas, Eastern Mediterranean, Europe, South-East Asia and Western Pacific	Case study analysing examples from other countries in funding public health and IHR (2005) implementation compared with the USA	N/A	1,3,4,5,7,8
Gheorghita & Caterinciuc, 2013^36^	Not reported	2013	Republic of Moldova	Europe	Case study of a national electronic reporting and surveillance system	N/A	1, 2, 3, 4, 5, 6, 7, 8
Hadjichristodouloua et al., 2013^38^	2008–2011	2013	European Union	Europe	Evaluation of a needs assessment project, a manual of European standards and best practices, and training materials and courses. Description of ship-to-port and port-to-port web-based communication networks and database for recording IHR ship sanitation certificates	8	1, 2, 3, 7
Kamradt-Scott et al., 2013^43^	2009–2010	2013	China, Japan, Republic of Korea and Thailand	South-East Asia, Western Pacific	Evaluation of contribution made by selected countries to the IHR (2005) revision process and how these governments are progressing in implementing revised IHR	N/A	1, 2, 3, 4, 5
Kasolo et al., 2013^46^	2012	2013	Africa	Africa	Assessment and report on Integrated Disease Surveillance and Response for building IHR (2005) core capacities	N/A	2, 3, 4, 6, 7, 8
Leventha et al., 2013^50^	2007–2013	2013	Israel, Jordan and West Bank and Gaza Strip	Eastern Mediterranean and Europe	Case study of the Middle East Consortium for Infectious Disease Surveillance	N/A	1, 2, 3, 5, 6, 7, 8
Rosewell et al., 2013^64^	2009–2012	2013	Papua New Guinea	Western Pacific	Evaluation of management of human resources in future health emergencies	N/A	1, 2, 7
Singh et al., 2013^77^	Not reported	Not reported	India (Andhra Pradesh)	South-East Asia	Case study and review of factors affecting outcomes of a surveillance project	8	2, 3, 8
Borchert et al., 2014^29^	2013	2014	Uganda	Africa	Case study of the rapid global health security enhancements targeting laboratory systems, information systems and coordination of information through emergency operations centres	N/A	1, 2, 3, 4, 5, 6, 7, 8
Hamblion et al., 2014^39^	2012–2014	2014	All overseas territories of the United Kingdom	Americas, South-East Asia and Europe	Evaluation of status of IHR (2005) compliance and appropriate measures to ensure compliance by June 2014	6	1, 2, 3, 4, 7, 8
Priotto et al., 2014^61^	2010–2011	2014	Morocco	Eastern Mediterranean	Evaluation of a strategy to strengthen the surveillance system and workforce by identifying technical capacities and training needs of public health officers	8	1, 2, 3, 7
Stewart-Evans et al., 2014^67^	Not reported	2013	European Union	Europe	Qualitative study of exposure assessment capabilities and communication pathways between exposure assessors and public health risk assessors	8	3
Wang et al., 2014^74^	2010	2014	China	Western Pacific	Evaluation of a multi-method training needs assessment, including reviews of competency domains needed to implement IHR (2005) as well as policies and emergency regulations	9	1, 2, 3, 4, 5, 7
Bekshin et al., 2015^76^	Not reported	2015	Kazakhstan	Europe	Case study of actions taken to improve surveillance and risk assessment	3	2, 3, 5, 6
Ihekweazu et al., 2015^41^	2011–2015	2015	South Africa and United Kingdom	Europe, Africa	Case study of a well supported collaboration between two public health institutes with similar mandates (Health Protection Agency of Public Health England collaboration with the National Institute for Communicable Disease)	N/A	1, 2, 3, 4, 7, 8
Lima & Costa, 2015^51^	2011	2015	Brazil	Americas	Case study of how IHR (2005) has been incorporated into the legal and administrative system	10	1, 2, 3, 4, 6, 7
Liu et al., 2015^52^	2014–2015	2015	China	Western Pacific	Evaluation of IHR (2005) accomplishments and opportunities	N/A	1, 2, 4, 5, 6, 7, 8
Standley et al., 2015^66^	Not reported	2015	Canada, Iraq, Kenya, Uganda, United Kingdom, and USA	Africa, Americas, Eastern Mediterranean and Europe	Evaluation of existing health security frameworks and the extent to which IHR (2005) and the Global Health Security Agenda overlap based on priorities for developing and executing biosecurity engagement programmes	8	1
Sylvester et al., 2015^65^	Not reported	2015	Burkina Faso, Cameroon, Kenya, Nigeria and Uganda	Africa	Evaluation of e-surveillance systems	6	1, 3
Ziemann et al., 2015^75^	Not reported	2015	Austria, Belgium, Denmark, France, Germany, Ireland, Italy, Spain, Sweden and United Kingdom	Europe	Evaluation of the benefits and pitfalls of syndromic surveillance	N/A	3

IHR: International Health Regulations; N/A: not applicable; WHO: World Health Organization.

^a^ Critical Appraisal Skills Programme qualitative research checklist summary score for eligible studies (see Table 2 for full quality assessment).^16^

^b^ IHR core capacities: 1 = national legislation; policy and financing; 2 = coordination and national focal point communication; 3 = surveillance; 4 = response; 5 = preparedness; 6 = risk communication; 7 = human resources; 8 = laboratory (Box 1).^7^

^c^ American Samoa (USA), Cook Islands, Federated States of Micronesia, Fiji, French Polynesia, Guam (USA), Kiribati, Marshall islands, Nauru, New Caledonia (France), Niue, Northern Marianas Islands (USA), Palau, Papua New Guinea, Samoa, Solomon Islands, Tokelau, Tonga, Tuvalu, Wallis and Futuna (France).

^d^ Cook Islands, Fiji, Kiribati, Nauru, Tuvalu, American Samoa (USA), Guam (USA), Palau, Papua New Guinea, Solomon Islands and Tonga.

^e^ Cook Islands, Federated States of Micronesia, Fiji, Kiribati, Marshall Islands, Nauru, New Caledonia (France), Northern Marianas Islands (USA), Niue, Palau, Papua New Guinea, Pitcairn Islands (United Kingdom), Samoa, Solomon Islands, Tonga, Tokelau, Tuvalu, Vanuatu and Wallis and Futuna (France).

**Table 2:** Quality assessment of studies included in the systematic review of the implementation of the International Health Regulations (2005)

Author, year	Aims clear	Qualitative methods suitable	Research design appropriate	Recruitment strategy appropriate	Data collection appropriate	Researcher–participants relationship considered	Ethical issues considered	Data analysis rigorous	Findings clear	Research of value	Total (out of 10)
Calain, 2007^30^	N/A	N/A	N/A	N/A	N/A	N/A	N/A	N/A	N/A	1	N/A
Lyons et al., 2007^53^	1	1	1	1	1	NS	NS	1	1	1	8
Narain et al., 2007^57^	N/A	N/A	N/A	N/A	N/A	N/A	N/A	N/A	N/A	1	N/A
Perry et al., 2007^60^	1	1	1	1	1	NS	NS	1	1	1	8
Kicman-Gawlowska, 2008^48^	N/A	N/A	N/A	N/A	N/A	N/A	N/A	N/A	N/A	1	N/A
Kicman-Gawlowska, 2009^49^	N/A	N/A	N/A	N/A	N/A	N/A	N/A	N/A	N/A	1	N/A
May et al., 2009^55^	N/A	N/A	N/A	N/A	N/A	N/A	N/A	N/A	N/A	1	N/A
Patel & Phillips, 2009^58^	N/A	N/A	N/A	N/A	N/A	N/A	N/A	N/A	N/A	1	N/A
Stikova et al., 2010^68^	N/A	N/A	N/A	N/A	N/A	N/A	N/A	N/A	N/A	1	N/A
Takahashi-Omoe & Omoe, 2009^70^	N/A	N/A	N/A	N/A	N/A	N/A	N/A	N/A	N/A	1	N/A
Van Vliet et al., 2009^72^	N/A	N/A	N/A	N/A	N/A	N/A	N/A	N/A	N/A	1	N/A
Chretien et al., 2010^31^	N/A	N/A	N/A	N/A	N/A	N/A	N/A	N/A	N/A	1	N/A
Johns & Blazes, 2010^42^	N/A	N/A	N/A	N/A	N/A	N/A	N/A	N/A	N/A	1	N/A
Kandun et al., 2010^44^	N/A	N/A	N/A	N/A	N/A	N/A	N/A	N/A	N/A	1	N/A
Kant & Krishnan, 2010^45^	N/A	N/A	N/A	N/A	N/A	N/A	N/A	N/A	N/A	1	N/A
Katz & Kornblet, 2010^47^	1	1	1	NS	1	NS	NS	1	1	1	7
Masanza et al., 2010^54^	N/A	N/A	N/A	N/A	N/A	N/A	N/A	N/A	N/A	1	N/A
Wamala et al., 2010^73^	1	1	1	1	1	NS	NS	1	1	1	8
Gomez et al., 2011^37^	1	1	1	1	1	NS	NS	1	1	1	8
Mmbuji et al., 2011^56^	N/A	N/A	N/A	N/A	N/A	N/A	N/A	N/A	N/A	1	N/A
Quandelacy et al., 2011^62^	N/A	N/A	N/A	N/A	N/A	N/A	N/A	N/A	N/A	1	N/A
Aguilera et al., 2012^26^	1	N/A	N/A	N/A	N/A	N/A	N/A	N/A	N/A	1	N/A
Gala et al., 2012^35^	1	1	1	1	1	NS	NS	1	1	1	8
Harutyunyan, 2012^40^	N/A	N/A	N/A	N/A	N/A	N/A	N/A	N/A	N/A	1	N/A
Kool et al., 2012^28^	1	1	1	1	1	NS	NS	1	1	1	8
Paterson et al., 2012^59^	1	1	1	1	1	NS	NS	1	1	1	8
Rajatonirina et al., 2012^63^	N/A	N/A	N/A	N/A	N/A	N/A	N/A	N/A	N/A	1	N/A
Teixeira et al., 2012^71^	1	1	1	1	1	NS	NS	1	1	1	8
Bakari & Frumence, 2013^27^	1	1	1	1	1	NS	NS	1	1	1	8
Craig et al., 2013^32^	N/A	N/A	N/A	N/A	N/A	N/A	N/A	N/A	N/A	1	N/A
Dagina et al., 2013^33^	1	1	1	1	1	NS	NS	1	1	1	8
Friede, 2013^34^	N/A	N/A	N/A	N/A	N/A	N/A	N/A	N/A	N/A	1	N/A
Gheorghita & Caterinciuc, 2013^36^	N/A	N/A	N/A	N/A	N/A	N/A	N/A	N/A	N/A	1	N/A
Hadjichristodouloua et al., 2013^38^	1	1	1	1	1	NS	NS	1	1	1	8
Kamradt-Scott et al., 2013^43^	N/A	N/A	N/A	N/A	N/A	N/A	N/A	N/A	N/A	1	N/A
Kasolo et al., 2013^46^	N/A	N/A	N/A	N/A	N/A	N/A	N/A	N/A	N/A	1	N/A
Leventha et al., 2013^50^	N/A	N/A	N/A	N/A	N/A	N/A	N/A	N/A	N/A	1	N/A
Rosewell et al., 2013^64^	N/A	N/A	N/A	N/A	N/A	N/A	N/A	N/A	N/A	1	N/A
Singh et al., 2013^77^	1	1	1	1	1	NS	NS	1	1	1	8
Borchert et al., 2014^29^	N/A	N/A	N/A	N/A	N/A	N/A	N/A	N/A	N/A	1	N/A
Hamblion et al., 2014^39^	1	1	NS	1	1	NS	NS	NS	1	1	6
Priotto et al., 2014^61^	1	1	1	1	1	NS	NS	1	1	1	8
Stewart-Evans et al., 2014^67^	1	1	1	1	1	NS	NS	1	1	1	8
Wang et al., 2014^74^	1	1	1	1	1	NS	1	1	1	1	9
Bekshin et al., 2015^76^	1	1	NS	NS	NS	NS	NS	NS	NS	1	3
Ihekweazu et al., 2015^41^	N/A	N/A	N/A	N/A	N/A	N/A	N/A	N/A	N/A	1	N/A
Lima & Costa, 2015^51^	1	1	1	1	1	1	1	1	1	1	10
Liu et al., 2015^52^	N/A	N/A	N/A	N/A	N/A	N/A	N/A	N/A	N/A	1	N/A
Standley et al., 2015^66^	1	1	1	1	1	NS	NS	1	1	1	8
Sylvester et al., 2015^65^	1	1	1	NS	1	NS	NS	NS	1	1	6
Ziemann et al., 2015^75^	N/A	N/A	N/A	N/A	N/A	N/A	N/A	N/A	N/A	1	N/A

N/A: not applicable; NS: not specified.

Notes: We made the quality appraisal using the Critical Appraisal Skills Programme qualitative research checklist.^16^ No studies were excluded on the basis of the quality assessment. Totals were not calculated for studies for which the criteria were not applicable.

Box 2 **Lessons learnt synthesized from the systematic review of national implementation of the International Health Regulations (2005), organized by core capacities 1–4**

**Core capacity 1: National legislation, policy and financing**

1. It is important to mobilize and sustain political commitment to developing the core capacities of IHR (2005) from central government, subnational levels of government, civil society and the scientific community.^40^^,^^59^

2. Developing appropriate policies and allocating responsibilities for the workforce is needed at both central and subnational levels of government.^29^^,^^39^^,^^61^

3. Legislation and policies should strive to incorporate and balance global, regional, national and subnational priorities for health security.^30^^,^^31^^,^^47^

4. For sustainable financing, detailed long-term national strategic plans with budget allocations should be developed in partnership between government, national stakeholders, and non-state actors (i.e. companies, high net-worth individuals and philanthropic foundations).^47^^,^^52^^,^^71^

5. The national health system and sociocultural context of a country will determine priorities for funding and other resource needs for IHR (2005) core capacity development, emergency response and the health workforce.^64^^,^^70^

6. Creating and empowering a national secretariat responsible for mobilizing and securing resources from state and non-state actors can help ensure sustainable and continuous financing.^39^^,^^44^^,^^47^

**Core capacity 2: Coordination and national focal point communications**

1. Existing regional and global intergovernmental organizations can be recruited to collaborate and co-manage international epidemics.^26^^,^^52^^,^^73^

2. A strong programmatic structure at the national and subnational levels, including intersectoral national technical working groups, can assist with comprehensive and effective coordination.^43^^,^^63^^,^^68^

3. Establishing roles, responsibilities and terms of reference of national focal points within national legislation, and disseminating information inside government, to national stakeholders, and to WHO, improves coordination and communication.^27^^,^^51^^,^^74^

4. Adequately resourced communication channels and standard operating procedures, from the subnational to the inter-governmental level, facilitate the timely transmission and retrieval of information.^26^^,^^29^^,^^57^^,^^73^

**Core capacity 3: Surveillance**

1. It is important to create and empower a national government entity to coordinate disease surveillance and control efforts.^26^^,^^27^

2. Strategic plans for disease surveillance at both the national and subnational levels can enable long-term sustainability of these efforts.^30^^,^^47^

3. Key communicable diseases need to be categorized based on epidemiological context (e.g. endemic diseases, target for disease elimination or eradication, epidemic-prone diseases and novel diseases).^26^^,^^30^^,^^60^

4. Surveillance at the subnational level can be sustained by developing legislation that outlines the requirements, mechanisms (e.g. indicator- or syndromic-based surveillance^7^) and frequency of reporting, stratified by communicable disease category and supported by guidelines and standard operating procedures.^33^^,^^38^

5. Validated training tools and resources (e.g. field epidemiology training programmes) can develop capacity for collection, analysis and use of data at subnational levels.^31^^,^^33^^,^^42^^,^^54^^,^^58^

6. A unified and dynamic surveillance system to track alerts, updates and early warnings and response, and using technologies appropriate to the national context (e.g. electronic, open-source, internet-based, real-time, mobile phone platform), encourages appropriate information use and accurate reporting.^28^^,^^33^^,^^37^

7. Standardizing passive data collection, management and validation procedures across all reporting sites, and actively searching for potential surveillance intelligence through local communication channels (e.g. community leaders, print media, internet, television), improves the usability and timeliness of data.^36^^,^^55^^,^^59^^,^^63^

**Core capacity 4: Response**

1. Defining criteria for an emergency public health event (e.g. seriousness, impact, unexpectedness, risk for international spread, risk for trade or travel) assists with the initiation of an appropriate and timely response.^36^^,^^39^

2. Establishing roles, responsibilities and terms of reference for a multidisciplinary response unit supports coordination of public health emergency responses at the national and subnational levels.^26^^,^^27^^,^^36^^,^^53^

3. Including national and international stakeholders in the development of guidelines for rapid and emergency response facilitates wide technical ownership and financial support.^39^^,^^46^^,^^52^

4. Designing and resourcing health facilities to minimize nosocomial infection transmission improves the efficacy of response without encouraging further spread (e.g. building isolation rooms, ensuring adequate ventilation, providing personal protective equipment).^26^^,^^73^

Box 3 **Lessons learnt synthesized from the systematic review of national implementation of the International Health Regulations (2005), organized by core capacities 5–8 and cross-cutting themes**

**Core capacity 5: Preparedness**

1. Comprehensive national and subnational budgeted plans for preparedness of emergency public health events are needed to support timely action.^26^^,^^27^

2. Exercises, drills, assessments and evaluations can test capacity to implement the response plans and ensure they are adequate and operational.^26^^,^^27^

3. A national reserve of medicines, vaccines and laboratory supplies and reagents helps with management of public health emergencies.^26^^,^^27^^,^^73^

**Core capacity 6: Risk communication**

1. An intersectoral system for risk communication of public health emergencies, functioning at all levels of government, ensures timely action and response.^33^^,^^46^^,^^59^

2. Risk communication systems should be bidirectional, with linkages to national and international public health partners, to collect and disseminate information about threats.^29^^,^^36^^,^^42^

3. Regular and emergency information to national and international stakeholders assists in building and maintaining effective communication channels and relationships.^28^^,^^45^^,^^73^

4. Standard operating procedures ensure personnel are ready to investigate and communicate emerging epidemics and support preparedness for activation of early warning systems.^26^^,^^42^^,^^57^

5. Using a combination of media to send and receive information ensures wide access and use.^33^^,^^35^^,^^46^^,^^68^

**Core capacity 7: Human resources**

1. Identifying competency gaps in the workforce ensures that training and resources are targeting the highest priority needs (e.g. epidemiology, problem-solving, management and leadership, technical expertise in pandemic and epidemic diseases).^54^^,^^61^^,^^74^

2. Selecting appropriate media for developing competencies based on health system context and social and cultural preferences can increase the efficacy of training.^38^^,^^45^^,^^61^

3. Establishing workforce oversight mechanisms for long-term maintenance of current and future needs assists with sustainability of individual training efforts.^30^^,^^41^^,^^44^

4. Nationally agreed strategies to recruit, train and retain health workforce can help support IHR (2005) competency development at all levels of the health system.^54^^,^^56^^,^^64^

**Core capacity 8: Laboratory**

1. It is important to create a governmental entity at the national level to coordinate national strategic plans for laboratory response and develop national standards, guidelines and operating procedures for laboratory surveillance at national and subnational levels.^26^^,^^34^^,^^52^^,^^54^^,^^73^

2. Regional and global reference laboratories can be helpful when developing laboratory surveillance networks.^27^^,^^41^^,^^42^

3. Validated criteria and mechanisms to accredit laboratories for core surveillance tasks help ensure high-quality testing.^31^^,^^54^^,^^73^

4. Laboratory training, routine assessments and feedback, and quality assurance systems can help improve surveillance capacity and function of national and subnational laboratories.^29^^,^^54^

5. Bidirectional flows of data, specimens and communication between national and subnational levels of government improve laboratory system effectiveness and adequate public health investigation.^27^^,^^29^

6. Specific policies, strategies, regulations and standard operating procedures for high-containment biosafety laboratories help ensure the safety and efficacy of laboratory testing.^29^^,^^52^^,^^73^

**Cross-cutting themes**

1. Conducting a baseline needs assessment can help to identify the status of a country’s health system and actions needed to meet IHR (2005) requirements.^27^^,^^54^^,^^73^

2. Ensuring flexible systems can improve response to known epidemics and detection of new threats.^29^^,^^70^^,^^74^

3. A country’s’ sociocultural, epidemiological, health system and economic context and priorities will guide national implementation of IHR (2005) core capacities.^59^^,^^64^^,^^68^

4. Although early warning systems are part of core capacity in surveillance, they also affect coordination, risk communication and response for new epidemics.^36^^,^^46^

5. Having a skilled workforce in place, from frontline staff to senior management, is important for ensuring sustainable expertise, implementation and commitment.^29^^,^^30^^,^^39^^,^^61^

IHR (2005): International Health Regulations, 2005 revision: WHO: World Health Organization. Notes: Framework for the analysis was based on the eight core capacities of the International Health Regulations (2005).^7^

### Implementation of core capacities

A total of 33 articles from 60 countries reported experience implementing the national legislation, policy and financing core capacity.^27^^,^^29^^–^^31^^,^^34^^–^^41^^,^^44^^,^^47^^,^^50^^–^^54^^,^^56^^,^^57^^,^^59^^–^^61^^,^ ^64^^,^^66^^,^^68^^,^^70^^–^^74^ In Cambodia, India and Uganda, the need to develop basic health-system functions (e.g. expanding access to health services that are essential for treating patients and for disease surveillance) had to be balanced against the commitment to meeting IHR (2005) requirements.^30^ Some low- and middle-income countries were concerned that large inflows of external funding for IHR (2005) would create vertical programmes that could not be fully incorporated within their health systems.^30^ In the USA, stakeholders need to ensure coverage and enforcement of legislation within private health-care facilities, where most citizens receive health services.^34^ In Brazil, creation of legislation, policy and guidelines was found to be insufficient without training and mechanisms to enforce implementation of IHR (2005).^51^

Experience implementing the coordination and communications capacity was reported in 33 articles from 51 countries.^26^^–^^31^^,^^33^^,^^36^^,^^38^^–^^43^^,^^46^^–^^48^^,^^50^^–^^52^^,^^56^^,^^57^^,^ ^59^^,^^61^^,^^63^^,^^64^^,^^66^^,^^68^^,^^70^^,^^71^^,^^73^^,^^76^^,^^77^ In Cambodia, India and Uganda, failing to coordinate through the national focal point led to duplication of surveillance strengthening efforts.^30^ In Papua New Guinea, capacity to govern is variable at the subnational level, and the reach of the national focal point needs to be expanded to local partners beyond the health sector.^33^

Forty articles from 63 countries reported experience implementing the surveillance core capacity.^26^^–^^43^^,^^45^^–^^47^^,^^50^^,^^51^^,^^53^^–^^55^^,^^57^^,^ ^59^^,^^60^^,^^62^^,^^63^^,^^65^^,^^67^^,^^68^^,^^71^^,^^73^^–^^76^ In the European Union, researchers identified a need for manuals of surveillance standards, guidelines, case definitions and protocols for reporting and disease notification.^38^^,^^39^ Reports from France, Iceland Indonesia and Madagascar noted the complementary nature of early warning systems, event-based surveillance and syndromic surveillance for detecting different types of epidemics.^55^^,^^63^^,^^75^ Experience in Papua New Guinea reinforced the need for periodic evaluations of syndromic and event-based surveillance systems.^33^ A study from the Pacific Islands identified a need to adapt surveillance information systems to the local context.^59^

Experience implementing the response core capacity was reported by 27 articles from 47 countries.^26^^–^^36^^,^^39^^,^^42^^,^^43^^,^^45^^,^^46^^,^^51^^–^^54^^,^ ^56^^,^^60^^,^^62^^,^^68^^,^^71^^,^^73^^,^^74^ Experience from the Republic of Moldova reinforced the importance of interdisciplinary rapid response teams (e.g. epidemiologists, microbiologists, hygienists, environmental health professionals and laboratory technicians).^36^ In Uganda, a command centre was created to house the rapid response team and to receive, evaluate and distribute information.^29^ Experience from Chile indicated the importance of having infection control committees composed of staff from different levels of the health system (e.g. hospital, provincial and national).^26^ A report from the United Kingdom of Great Britain and Northern Ireland and its territories emphasized the need for response plans to be reviewed and updated regularly to respond to all hazards: zoonotic, food safety, chemical, radionuclear and antimicrobial resistance threats.^39^

Thirteen articles from 37 countries reported experience implementing the preparedness core capacity.^26^^,^^27^^,^^29^^,^^32^^,^^34^^–^^36^^,^^40^^,^^45^^,^ ^49^^,^^50^^,^^52^^,^^56^^,^^73^^,^^74^^,^^76^ In the United Republic of Tanzania, failure to create specific budget lines for public health preparedness hampered preparedness planning.^27^ Experience from Asia during the H1N1 influenza pandemic found that, in addition to national plans, regions may need to develop multicountry preparedness plans focused on real-time information sharing.^50^

A total of 23 articles from 36 countries reported on implementing the risk communication core capacity.^26^^–^^29^^,^^33^^,^^35^^,^^36^^,^^42^^,^^45^^,^^46^^,^^50^^–^^53^^,^ ^55^^,^^57^^,^^59^^,^^68^^,^^71^^,^^73^^,^^76^ Experience from Papua New Guinea and the USA found that regular interactions with the media helped to build relationships, trust and functioning communication streams for use in times of emergency.^33^^,^^42^ In the Republic of Moldova and sub-Saharan Africa, bi-directional communication streams allowed central authorities to communicate with subnational levels to raise awareness, receive information from subnational authorities, and ultimately conduct investigations on disease epidemics.^36^

A total of 37 articles from 49 countries reported experience implementing the human resources core capacity.^26^^,^^27^^,^^29^^–^^31^^,^^33^^–^^39^^,^^41^^,^^42^^,^^44^^–^^46^^,^^49^^–^^54^^,^^56^^–^^59^^,^^61^^–^^64^^,^^68^^,^^71^^–^^74^ Experience from Cambodia, India, Uganda and the United Kingdom indicated that traditional curricula, competencies and training did not prepare the workforce to implement IHR (2005) and that additional knowledge transfer and skill-building is needed to ensure reporting and data use at subnational levels.^30^^,^^39^ The sociocultural context influenced learning preferences; for example, in Morocco face-to-face learning was preferred, while in India and the United Kingdom electronic learning was preferred.^39^^,^^45^^,^^61^ In China, Morocco, South Africa and the United Kingdom, interactive and skill-building sessions were preferred over static knowledge transfer.^41^^,^^61^^,^^74^ Settings that had high staff turnover (e.g. rural areas, those with armed conflicts) faced staff shortages and required unique mechanisms for continual retraining.^31^^,^^39^^,^^59^^,^^63^

Experience implementing the laboratory core capacity was reported in 26 articles from 44 countries.^26^^–^^31^^,^^34^^,^^36^^,^^39^^–^^42^^,^^45^^,^^46^^,^^50^^,^ ^52^^–^^54^^,^^56^^,^^57^^,^^62^^,^^68^^,^^71^^,^^73^^,^^77^ Studies in Afghanistan, Uganda and the United Kingdom identified a need to develop laboratory capacity for basic tasks such as collection, transportation and analysis of specimens.^31^^,^^39^^,^^73^ Several sub-Saharan counties needed laboratory accreditation systems, supported by training and mentoring, and external quality assurance systems in place, as well as laboratory information systems that directly influence public health action.^54^

### Cross-cutting themes

We identified five global lessons learnt that related to multiple IHR (2005) core capacities (Box 2). Some major cross-cutting themes included the need for mobilizing and sustaining political commitment; for adapting global requirements based on the local sociocultural, epidemiological, health system and economic contexts; and for conducting baseline and follow-up assessments to monitor IHR (2005) status.

## Discussion

We found substantial documentation of lessons learnt from implementing IHR (2005) core capacities globally. Although many factors affected implementation in a country, the structure of the health system, sociocultural factors, economic status and type of governance, we were able to extract some common themes. We found few gaps in implementation in direct relation to the IHR (2005) core capacities. Nonetheless, additional research could be useful in evaluating countries’ core capacity performance against actual disease epidemics. Specifically, we did not identify any documentation evaluating IHR (2005) core capacity development with national performance in preventing, detecting and responding to disease epidemics. This type of evaluation could provide useful data for future revisions of the IHR. Moreover, defining fundamental data needs for new treatments and vaccines could help develop national research capacity to shorten the development window during future disease epidemics. Finally, additional documentation of experience from Eastern Europe and Africa could help fill knowledge gaps.

Countries had different national health plans outlining how to organize their systems and strategies for managing threats and hazards.^78^ National plans typically outlined how health priorities will be managed and linked to programme-specific strategic plans, such as those for primary health care, maternal and child health, human immunodeficiency virus and acquired immune deficiency syndrome (HIV/AIDS). To avoid having only vertical programmes, countries will have to decide how to integrate IHR (2005) core capacity development within existing strategic plans, such as those for the health workforce or laboratory systems. Integration of IHR (2005) into national legislation is also required. To achieve this, WHO proposed that countries need to consider how the IHR (2005) are to be implemented based on their national legal and governance context; to assess existing legislation for IHR (2005) implementation; to revise existing legislation or adopt new legislation; and to enforce nationally adapted legislation.^69^^,^^79^

Most countries reported some experience in implementing communicable disease surveillance as part of IHR (2005).^80^ Although core capacity requirements for surveillance are the same for all countries, the means to achieve them differed. For example, real-time systems worked well in settings with good electronic and telecommunications infrastructure, while delays were common in settings with more limited infrastructure.^59^ Such delays were acute in rural settings where health staff face logistic challenges in reporting events and may not be fully aware of the benefits of reporting events.^33^ Nevertheless, minimizing delays in discovering and declaring disease outbreaks remains a global priority.^81^ Prospectively, vertical disease surveillance systems (e.g. population-based surveys for HIV/AIDS, case reporting for malaria and cohort monitoring for tuberculosis) could be used and adapted when developing and maintaining the surveillance core capacity of IHR (2005).^30^

Although complementary in nature, the preparedness and response core capacities were often implemented independently of one another. For example, areas requiring coordination across both of these core capacities, such as case management or infection control, were not well-documented. Taking a holistic approach to IHR (2005) core capacity development could avoid these issues.^26^^,^^45^^,^^52^ Moreover, there were cases in which it was difficult to implement national IHR (2005) policies. For example, in the United Republic of Tanzania protocols for isolating patients with certain diseases were developed, but due to shortages of public-sector health facilities private sector hospitals had to be used during epidemics.^27^ This highlights the need to adapt global core capacities requirements to locally appropriate policies and solutions.

Risk communication focuses primarily on effective communication strategies during disease epidemics. Some of the articles we identified primarily discussed communication systems.^33^^,^^46^^,^^59^ For example, while many countries documented positive experience with internet-based communication strategies, inconsistent access to electricity and telecommunications in the United Republic of Tanzania made this challenging.^27^ More documentation of risk communication lessons learnt during epidemics are needed. For example, there was delayed notification and underreporting of cases from the national to international level during the severe acute respiratory syndrome epidemic in China in 2002.^16^ This delay may have contributed to preventable transmission of the virus and galvanized international interest in revising IHR(2005) to ensure transparency and timeliness of communication during disease epidemics.^82^^,^^83^

The World Health Assembly recently approved the *Global strategy on human resources for health: workforce 2030*.^84^ Development of human resources required by IHR (2005) could be included within national strategies for human resource development. Initial quantification of needs could be completed through the use of WHO recommended tools such as the Workload Indicators of Staffing Need.^85^ Most health workforce strategies are long-term and require development and revision of curricula, competencies, professional continuing education and licensing. Although international support plays a role in training local staff, Indonesia and South Africa, among others, noted that this mechanism was often resource-intensive and unsustainable.^41^^,^^42^^,^^44^ Moreover, given that front-line health staff may be resistant to additional administrative duties and responsibilities without increased remuneration, it is essential to reduce redundancies in roles and responsibilities across different disease programmes.^30^

Laboratory networks are an essential part of national and regional health systems. Global laboratory networks are in place for polio, immunization programmes and influenza.^86^^–^^88^ It could be worthwhile to adapt and integrate these existing networks to meet IHR (2005) requirements. The issue of limited capacity to develop laboratory infrastructure in rural areas was raised.^39^ In these settings, disease-specific point-of-care assays may be needed, even if they do not contribute to overall strengthening of the laboratory system. Decentralized laboratory assays that contribute to control of multiple diseases (e.g. nucleic acid amplification that could be used for HIV/AIDS, tuberculosis, viral hepatitis, novel influenza strains and Ebola virus disease), rather than disease-specific assays, may be a better way to strengthen laboratory systems.^89^

Given the many priorities in development, and limited national budgets to achieve them, external funding opportunities often help mobilize political commitment and national action. Experience from South Africa suggested that while external funding sources can be inflexible and unsustainable within national governance frameworks, such funding could create momentum in generating domestic financing and accelerate progress in implementing IHR (2005).^41^ Therefore, identifying adequate funding is the first step to developing, strengthening and maintaining national IHR (2005) core capacities. Global eradication of polio and global elimination of HIV/AIDS, tuberculosis and malaria were all costed and have been successful in mobilizing political commitment.^90^^–^^93^ Estimating the global and national costs of meeting IHR (2005) core capacities could also help in identifying external funding sources. Various frameworks and tools are available to cost IHR core capacity development.^94^^,^^95^ Depending on the setting and context, the World Bank, the International Monetary Fund, regional development banks, bilateral partners and non-state actors (i.e. companies, high net-worth individuals and philanthropic foundations) could all make substantial contributions towards advancing global implementation of IHR (2005).^96^^–^^101^

Along with financial and technical support, additional international measures could help accelerate IHR (2005) core capacity development. For example, since past disease epidemics affected regional and global economic growth, economic issues are relevant.^102^^,^^103^ Allowing countries that meet IHR (2005) core capacity requirements to participate in regional and global trade agreements, or have access to grants or low-interest loans through bilateral partnerships or international financing institutions, could help mobilize political commitment and action. Conversely, if there remain persistent challenges in meeting the agreed IHR (2005) core capacity deadlines, trade tariffs, sanctions or embargoes might be needed to ensure countries collaborate fully in sustaining global health security. These different measures could also be used during disease epidemics in response to countries’ compliance or lack of compliance with IHR (2005) requirements. This could help, for example, accelerate the timeliness of providing essential surveillance data to the global health community. This would represent a shift in current enforcement of IHR (2005) core capacity development that may require discussion and consensus outside of the health sector.^104^ Regardless of whether macroeconomic measures are implicated with IHR (2005), failure to implement IHR (2005) core capacities could adversely affect the international image of a country and also increase its susceptibility to economic losses due to disease epidemics.

This study has several limitations. We focused only on IHR (2005) core capacities and not on the core capabilities of points of entry, zoonotic events, food safety, chemical events and radiation emergencies.^105^ Future studies should evaluate these additional aspects of IHR (2005). The databases we used only have the capacity to search the titles and abstracts of articles and may have missed articles that only mentioned IHR-related keywords in the full text. Using databases that have the capacity to search the full text may identify additional studies.^106^ There is little consensus on the use of quality appraisal in qualitative meta-ethnography and we therefore did not exclude any qualitative studies based on quality rating.^18^ In addition, given the limited availability of formal qualitative research, we included all documentation related to IHR (2005) irrespective of study design. We relied on publicly available documentation from countries; some countries or institutions may not have published their experiences in implementing IHR (2005) due to competing priorities, limited capacity or lack of awareness on the importance of sharing their experience. After completion of this systematic review, Joint External Evaluation guidance became publicly available.^107^ Their mission reports provide valuable information that should be included in updates to this review.^108^ Furthermore, annual submissions on IHR (2005) from Member States to the World Health Assembly likely contain relevant information, but are not publicly available.

Despite considerable progress, countries that are yet to implement IHR (2005) core capacities may have insufficient human and financial resources to meet their obligations in the near future. Recent global epidemics have galvanized high-level political commitment towards ensuring global health security. We can leverage this commitment by mobilizing resources and securing wider collaboration to apply the lessons outlined here and accelerate the development of IHR (2005) core capacities globally.
